# The Power of School Conditions: Individual, Relational, and Organizational Influences on Educator Wellbeing

**DOI:** 10.3389/fpsyg.2022.775614

**Published:** 2022-03-09

**Authors:** Rachel Fiona Cann, Claire Sinnema, Alan J. Daly, Joelle Rodway, Yi-Hwa Liou

**Affiliations:** ^1^Faculty of Education and Social Work, The University of Auckland, Auckland, New Zealand; ^2^Department of Education Studies, University of California, San Diego, San Diego, CA, United States; ^3^Faculty of Education, Memorial University of Newfoundland, St. John’s, NL, Canada; ^4^Educational Management, National Taipei University of Education, Taipei, Taiwan

**Keywords:** positive psychology, social network analysis, subjective wellbeing, psychological wellbeing, educator wellbeing, positive education

## Abstract

Wellbeing in schools is often focused at the individual level, exploring students’ or teachers’ individual traits, habits, or actions that influence wellbeing. However, studies rarely take a whole-school approach that includes staff wellbeing, and frequently ignore relational and organizational level variables. We take a systems informed positive psychology approach and argue that it is essential to build greater understanding about organizational and relational influences on wellbeing in order for schools to support educator wellbeing. Our study evaluated the relative contributions of individual, relational, and organizational factors to educator wellbeing. Our measure of wellbeing focused on the life satisfaction and flourishing of 559 educators in 12 New Zealand schools. We used a social network analysis approach to capture educators’ relational ties, and demographic data and psychometric scales to capture individual and organizational level variables. Results of hierarchical blockwise regressions showed that individual, relational, and organizational factors were all significantly associated with educator wellbeing; however, it was educators’ perceptions of trusting and collaborative school conditions that were most strongly associated with their wellbeing. The number of relational ties educators had explained the least amount of variance in wellbeing. Educators were more likely to experience high levels of support when their close contacts also experienced high levels of support. However, for many educators, there was a negative association between their most frequent relational ties and their reported levels of support. Our results suggest that attending to the organizational factors that influence wellbeing, through creating trusting and collaborative school conditions, may be one of the most influential approaches to enhancing educator wellbeing. We call for whole-school approaches to wellbeing that not only consider how to support and enhance the wellbeing of school staff as well as students, but also view the conditions created within a school as a key driver of wellbeing within schools.

## Introduction

An abundance of research shows that teachers suffer from high rates of stress and depression (Milfont et al., 2008; Yang et al., 2009; Kidger et al., 2010; Greenberg, 2016), and this has negative consequences for students’ academic achievement and wellbeing (Roffey, 2012; Hoglund et al., 2015). Although some studies identify teaching as more stressful than similar professions (for example: Worth and Van den Brande, 2019), this is debated as ambulance workers, prison officers, and police have been shown to experience stress levels as high, or higher, than teachers (Johnson et al., 2005). Positive psychology looks beyond stress and depression (the negative end of the wellbeing spectrum) and seeks to “understand and build the factors that allow individuals, communities, and societies to flourish” (Seligman and Csikszentmihalyi, 2000, p. 5). This sentiment is taken up by the positive education movement, which applies ideas from positive psychology to the work of schools in improving students’ wellbeing alongside their academic outcomes. Over the last 10 years, schools are increasingly adopting positive education approaches, yet teacher wellbeing is often neglected in the quest to improve student wellbeing, and positive education has been critiqued for a lack of studies into whole-school approaches to wellbeing that include the promotion of staff wellbeing (Waters and Loton, 2019). The purpose of this study is to explore educator wellbeing through a positive psychology lens to uncover the factors that may positively influence educator wellbeing, rather than simply reducing educator stress.

Research has revealed a number of practices that can boost wellbeing—for example, mindfulness (Lomas et al., 2019) or gratitude (Davis et al., 2016)—and circumstances that can positively or negatively influence wellbeing, such as standard of living (Eger and Maridal, 2015) or workplace culture (Sojo et al., 2016). However, there is debate about the relative influence of different determinants of wellbeing, and the degree to which individuals can exercise agency in their wellbeing. The frequently cited “happiness pie” suggests that 50% of happiness is determined by genetic factors, 40% by individuals’ intentional activities, and 10% by individuals’ circumstance (Lyubomirsky et al., 2005). Follow-up work by the same authors suggest the link between an individual’s intentional actions and their wellbeing may be weaker than initially believed (Sheldon and Lyubomirsky, 2021), and some critics have suggested that an individual’s intentional activities could account for as little as 5% of the variation in wellbeing (Brown and Rohrer, 2019). However, the majority of research within positive psychology focuses on the individual and ignores their broader social context (Kern et al., 2020), a stance that has been increasingly critiqued from both outside and within the field. Systems Informed Positive Psychology (SIPP) incorporates principles from the systems sciences—such as interconnectedness, dynamics, and multiple perspectives—and has been proposed as an approach to ensure that contextual factors are considered in wellbeing research (Kern et al., 2020).

In order to explore how relationships with others influence wellbeing, and in keeping with the SIPP approach, we use social network theory. The importance of relationships and social capital is central to social network theory. Social capital is generally defined as the existing and potential resources embedded within networks of relationships individuals can access and mobilize for purposive actions (Nahapiet and Ghoshal, 1998). The application of social network analysis in educational settings has shown that schools’ social networks can impact innovation (Liou and Daly, 2018), the improvement of teaching practice (Sinnema et al., 2021), curriculum and policy implementation (Coburn and Russell, 2008; Hopkins et al., 2017), and student achievement (Daly et al., 2014). Within the workplace, social capital is significantly associated with employee wellbeing (Berraies et al., 2020). Central to the definition of social capital are key aspects that include: structural aspect, relational aspect, and cognitive aspect (Nahapiet and Ghoshal, 1998; Liou and Canrinus, 2020). The structural aspect of social capital concerns the pattern of relationships between individuals that can support and/or hinder the flow of resources necessary for purposive actions (Lin, 2001). The relational aspect of social capital refers to the quality of relationships that may affect the speed, depth, and complexity of resources traveled among individuals (Granovetter, 1992; Nahapiet and Ghoshal, 1998). The structural and relational aspects of social capital are considered in our exploration of the relational factors that influence wellbeing. The third aspect, the cognitive aspect of social capital, deals with individuals’ tendency to interact with others based on their interpretations and beliefs about the knowledge, norms, or specialized discourse that they experience through interactions (Moolenaar et al., 2014; Liou and Canrinus, 2020). We consider the cognitive aspect of social capital in the exploration of individual factors that influence wellbeing.

Also of particular interest in this research is positive social capital which expands the generative capacity of people and helps them to flourish (Baker and Dutton, 2017). Positive social capital can be created through organizational practices that build trust, enable collaboration, and value relational skills (Baker and Dutton, 2017). Rather than focus on educators’ perceptions of trust and collaboration at the dyadic level, we examine educators’ perceptions of trusting and collaborative school conditions as part of the organizational factors influencing wellbeing.

Overall, using the SIPP approach in our study, we examine how educators’ wellbeing is influenced by factors at three distinct levels: the individual, the relational, and the organizational.

### Wellbeing

Research into educator wellbeing is generally focused on teachers, rather than all educators (for example, support staff are generally not included). Studies often focus on teacher stress and depression and seldom define what teacher wellbeing means (McCallum et al., 2017). Where teacher wellbeing is defined, these definitions vary widely. McCallum and Price (2016) describe the nature of teacher wellbeing as “diverse and fluid… unique to each of us” (p. 17), whereas the OECD proposes that teacher wellbeing comprises four core dimensions: cognitive wellbeing, subjective wellbeing, physical and mental wellbeing, and social wellbeing (Viac and Fraser, 2020).

From a positive psychology perspective, wellbeing is more than the absence of negative states, such as stress or depression—wellbeing is characterized by various indicators, such as positive emotion, meaning in life, and feelings of satisfaction. Wellbeing has been conceptualized and measured through different lenses, for example: subjective wellbeing, psychological wellbeing, and social wellbeing (Disabato et al., 2021). However, a number of studies have noted that measures of wellbeing tend to overlap; for example, self-report measures of subjective wellbeing and psychological wellbeing have shown a correlation of 0.96, and other measures of wellbeing based on models of emotional or social wellbeing also show large correlations (Goodman et al., 2020). A hierarchical model of wellbeing has been proposed where different lenses on wellbeing (such as subjective wellbeing) sit underneath general wellbeing, which is “defined as *perceived enjoyment and fulfillment with one’s life as a whole*” (Goodman et al., 2020, p. 3 emphasis in original). We use a broad conceptualization of wellbeing which is grounded in positive psychology, using measures of subjective wellbeing and psychological wellbeing (i.e., the Flourishing scale, see Diener et al., 2010), in an attempt to capture *educators’ perceived enjoyment and fulfillment with their work as an educator*.

A number of mechanisms that link various workplace factors to employee wellbeing have been identified through research that applies the job demands-resources model (Xanthopoulou et al., 2007; Jourdain and Chênevert, 2010; Yin et al., 2018). Job demands include factors, such as workload, emotional demands, and role stress, while resources include social support, autonomy, and professional development (Xanthopoulou et al., 2007; Jourdain and Chênevert, 2010). Xanthopoulou et al. (2007) find that the supply of job resources is associated with greater work engagement and lower levels of exhaustion. They explain that an underlying psychological mechanisms of this association is the activation of employees’ personal resources of self-efficacy, self-esteem, and optimism (Xanthopoulou et al., 2007). Jourdain and Chênevert’s (2010) study of nurses found that job demands were associated with reduced professional commitment and a greater intention to leave the profession. They suggest the mechanisms underlying this association are a gap between nurses’ aspirations and their actual work performance, and a lack of identification with the profession when nurses doubt they are suited to the profession (Jourdain and Chênevert, 2010). Workplace factors and employees’ personal resources and perceptions are considered in this study as we measure the association between individual, relational, and organizational factors, and educator wellbeing.

### Individual Factors and Wellbeing

Social capital has frequently been associated with greater levels of wellbeing (Helliwell and Putnam, 2005; Klein, 2013). The cognitive aspect of social capital posits that individuals possess a certain level of cognitive perceptions of their surrounding social networks that may affect their decisions to assess the quality and make use of their personal networks (Krackhardt, 1987). In this regard, individuals are cognizant of their personal networks and are intentional about their decisions as to the development of their networks as they access and mobilize social capital for purposive actions (Burt and Ronchi, 2007). Individuals being intentional about personal networks also mean they are knowledgeable of the value of their social contacts and the level of social influence that may have on them (Borgatti and Cross, 2003). Individuals who are aware of their networks of relationships are more likely to attend to the quality and purpose of their networks, and as such they are more likely to obtain social support that may lead to overall wellbeing (Thoits, 2011). Individuals also assess the appropriateness of their own beliefs and behaviors against the norms of group in which they are embedded (Thoits, 2011). Thus individuals’ wellbeing can be positively or negatively impacted by social influence in that their beliefs, behaviors, or habits may be affected by the norms of their network contacts, such as proactive or poor habits (Cohen, 2004).

In general, studies have shown that cognitive social capital is associated with greater wellbeing. Cognitive social capital (conceptualized as trust, reciprocity, and mutual help) was significantly associated with subjective wellbeing in a rural Chinese population (Yip et al., 2007), and for social networking site users, their cognitive social capital (conceptualized as shared goals and values) was significantly associated with subjective wellbeing (Chang and Hsu, 2016). However, in the workplace, Berraies et al. (2020) found that cognitive social capital (conceptualized as shared vision, goals, and values) was not significantly associated with employee wellbeing. While these studies used various conceptualizations to measure cognitive social capital, in our study, we use the concept of *network intentionality*—the extent to which an educator is intentional in shaping their personal networks. Given that previous studies generally show a positive association between cognitive social capital and wellbeing, we propose that network intentionality would be positively associated with wellbeing.

At the individual level, a number of demographic variables may be associated with wellbeing: years as an educator, role within the school, and gender, in particular. The longer teachers remain in the profession, the more likely they are to report greater positive affect and less burnout (Gloria et al., 2013). In contrast, up to 50% of teachers leave the profession in the first 5 years (Arnup and Bowles, 2016), many citing workload as one reason, and 51% also stating that teaching was making them ill (Perryman and Calvert, 2020). Overall, the number of years an educator remains in the profession has been positively associated with wellbeing. An individual’s position within formal hierarchies at work has been shown to influence wellbeing. People further down the hierarchy have less control over their work and, therefore, experience more stress, while those in leadership positions experience less stress (Marmot, 2004). However, leadership positions within schools are often viewed as more stressful than teaching roles, with many New Zealand teachers reluctant to move into middle leadership roles (such as head of department) due to the associated workload and pressure (Cameron et al., 2007), and 59% of primary principals reporting high to extremely high levels of stress (Wylie and MacDonald, 2020). As the context of this study is a school, we proposed that people with leadership roles were likely to have lower levels of wellbeing than other educators. Finally, gender was also included in the demographic variables. Studies that explore how gender impacts employee wellbeing show that women report lower wellbeing (Ravenswood et al., 2017) and female teachers experience higher levels of workload stress and classroom stress (Klassen and Chiu, 2010).

### Relational Factors and Wellbeing

Social interactions are associated with wellbeing (Sandstrom and Dunn, 2014), and these can be explored using the structural aspect of social capital, which concerns the pattern of relationships embedded within networks. Relational ties between people enable the flow of resources, and the patterns of these ties form structures that can enable or constrain action (Wasserman and Faust, 1994; Cross and Parker, 2004). Patterns of relational ties have been explored in relation to wellbeing. The more people that a person interacts with on a given day, the greater their reported levels of happiness, even when the interactions are with weak ties (i.e., individuals a person does not know well or feel close to; Sandstrom and Dunn, 2014). The greater a person’s social activity, the greater their levels of positive affect (Watson et al., 1992) and happiness (Sun et al., 2019). Yet, social network analysis is seldom used to explore educator wellbeing. Therefore, we use social network analysis to explore the links between educators’ interactions within their network and their wellbeing.

Relational social capital concerns relationships that can influence an individual’s behavior (Nahapiet and Ghoshal, 1998). In terms of wellbeing, the people to whom an individual is connected in a network can influence that individual’s wellbeing through their affect and actions. For example, an individual’s happiness influences the happiness of those in their social network (Fowler and Christakis, 2008), and prosocial behavior in the workplace has been shown to propagate through networks (Chancellor et al., 2018). Therefore, we also consider the possible influences of an individual’s connections through the idea of *network exposure* (the wellbeing of the people an individual is connected to) and explore any connections between wellbeing and the informal communities that are formed within networks.

### Organizational Factors and Wellbeing

People’s perception of social climate is associated with their wellbeing, for example, levels of trust in a society are shown to be associated with subjective wellbeing (Helliwell and Wang, 2010). Within workplaces, organizational conditions are significant predictors of wellbeing, even when controlling for individuals’ personality traits (Parent-Lamarche and Marchand, 2019), and relational culture explains variance in wellbeing above that predicted by personality traits (Di Fabio et al., 2016). Organizational research identifies values, such as trust, support, and collaboration that underpin cultures that increase aspects of wellbeing, such as employee satisfaction and commitment (Schneider et al., 2013). Schools with a positive culture and good social support help to reduce teacher stress (Kyriacou, 2001). When teachers have trusting relationships with others in school, they have lower levels of burnout, and lower levels of both anxiety and depression (Van Maele and Van Houtte, 2015; Yin et al., 2018; Huang et al., 2019). Teachers also cite collaborative working relationships with colleagues as contributing to developing confidence, reducing anxiety, and enhancing their wellbeing (Paterson and Grantham, 2016) and that collaborative relationships can help to offset some of the negative aspects of teaching (Wigford and Higgins, 2019). The importance of cultures of trust and collaboration is well established in education, as they are linked to teacher learning and school improvement (e.g., Bryk and Schneider, 2002). In our study, we explore the links between trusting and collaborative school conditions and educator wellbeing. For these organizational level factors, we focus on educators appraisals of the general school conditions of trust and collaboration, and view this as distinct from relational level factors which focus on the quality of particular relationships at the dyadic level.

### The Present Study and Hypotheses

Using a systems informed positive psychology lens, we explored how wellbeing is associated with different individual, relational, and organizational factors, including the relative contributions of each of these factors to educator wellbeing. Based on the empirical research described, the following hypotheses were formulated.

At the individual level:

*Hypothesis* 1: An individual’s level of network intentionality will be positively associated with wellbeing.

*Hypothesis* 2: Demographic variables will be associated with wellbeing: (a) years of experience will be positively associated with wellbeing, (b) having a leadership role will be negatively associated with wellbeing, and (c) being female will be negatively associated with wellbeing.

At the relational level:

*Hypothesis* 3: The number of relational ties an educator has will be positively associated with their wellbeing.

*Hypothesis* 4: The positive resources available to educators through their connections (such as high wellbeing or support provided) will be positively associated with wellbeing: (a) the wellbeing of the people an individual has relational ties with will be positively associated with that individual’s wellbeing and (b) the informal community to which an individual belongs will be associated with wellbeing (either positively or negatively depending on the nature of the community).

At the organizational level:

*Hypothesis* 5: An individual’s perception of trusting and collaborative school conditions will be positively associated with the individual’s wellbeing.

## Materials and Methods

### Data Collection and Sample

The study was conducted in 12 schools in New Zealand (nine elementary and three secondary schools). The schools were members of two “Communities of Learning” (CoLs), which comprise a group of schools, typically in close proximity, working together to define and address achievement challenges they share. Sampling rules that enable the evaluation of the reliability of a sample drawn from a population do not apply when judging the quality of relational data derived from a sample (Scott, 2000). The structure of a sample network may not be representative of the structure of the network from which it is drawn (Scott, 2000). Therefore, a common approach within social network studies is to use a bounded-saturated approach (or full network census) where all members of an identified group are included (Lin, 1999; Scott, 2000; Hanneman and Riddle, 2005; Borgatti et al., 2018). This approach is a standard method in social network research in schools, for example, in studies of: advice seeking (Sinnema et al., 2021), data use (Farley-Ripple and Buttram, 2015), and curriculum implementation (Hopkins et al., 2017). This strategy, coupled with high response rates, produces more valid information as it provides a more complete picture of the relations within the network (Scott, 2000; Hanneman and Riddle, 2005). In this study, two groups were identified—the two CoLs—and all educators were included in the study. This case study approach allows us to understand the factors affecting educator wellbeing for the population of the two CoLs. Educators—including teachers, classroom assistants, curriculum leaders, and principals—were surveyed to collect data about their social networks, perceptions about the nature of their relationships and school conditions, and demographic information. A total of 559 educators were invited to participate, and 469 (84%) completed the survey (see section “Descriptive Statistics” and Table 1 for descriptive data).

**Table 1:** Descriptive data.

	Frequency	%	*N*	Minimum	Maximum	Mean	Std. deviation
*Demographics*							
Gender							
Female	342	72.9					
Male	120	25.6					
Non-binary/prefer not to say	7	1.5					
Years working as an educator			426	0	50	15.2	12.2
Formal role							
Teacher aides	41	8.7					
Teachers	220	46.9					
Middle leaders	167	35.6					
Senior leaders	41	8.7					
*Scales*							
Wellbeing							
Satisfaction			449	1.50	6.00	4.62	0.74
Contribution			449	1.60	6.00	5.42	0.50
Support			449	2.50	6.00	4.94	0.60
Network intentionality							
Seeking			421	1.75	6.00	4.60	0.85
Beliefs			421	2.67	6.00	5.22	0.65
Assessment			421	1.00	6.00	3.87	1.17
Connect			421	1.25	6.00	4.57	0.80
Trusting relationships			441	1.71	6.00	4.95	0.66
Resources for collaboration			432	2.00	6.00	4.61	0.72
Social network statistics							
Close relationship outdegree (normalized)			469	0.000	0.341	0.034	0.037
Close relationship indegree (normalized)			469	0.000	0.093	0.030	0.018
Advice (monthly) outdegree (normalized)			469	0.000	0.290	0.042	0.049
Advice (monthly) indegree (normalized)			469	0.000	0.162	0.038	0.029
Close relationship outdegree			469	0	103	9.6	10.8
Close relationship indegree			469	0	28	8.49	5.4
Advice (monthly) outdegree			469	0	81	12.1	14.4
Advice (monthly) indegree			469	0	49	11.0	8.9

The study was reviewed and approved by the research ethics committee at the corresponding author’s institution. Informed consent was obtained from principals of the 12 schools in order to invite staff at their schools to participate in the research. An online questionnaire was sent to 559 educators across the 12 schools in March 2020.

### Variables

**Figure 1:**
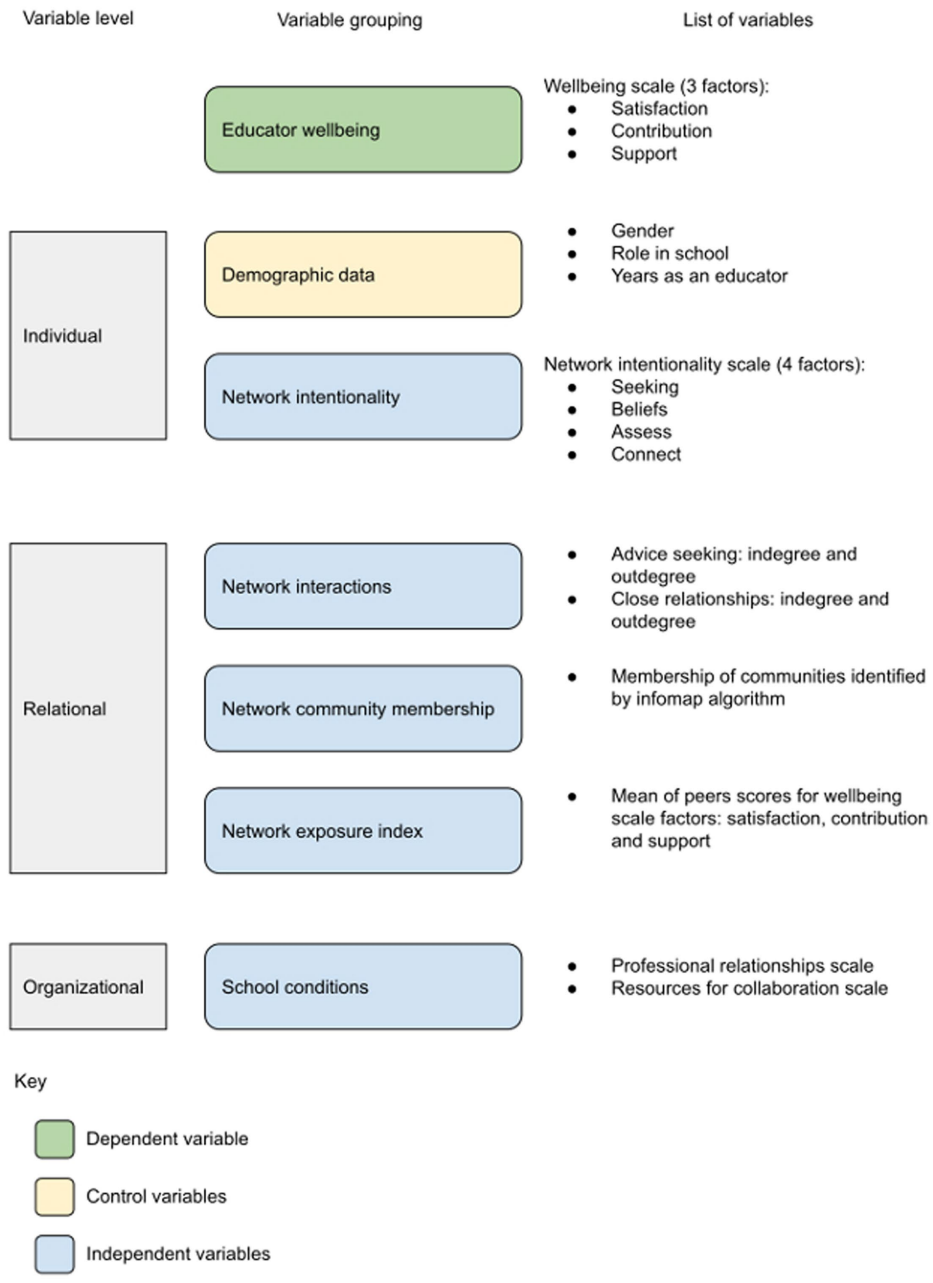
Variables used in this study.

The variables were grouped into individual, relational, and organizational factors, as shown in Figure 1. The questionnaire included four scales to measure educators’ wellbeing, network intentionality, and their perceptions of trusting relationships and collaboration within their schools. Each scale has been validated in prior research, and the wellbeing scale was adapted from two previously validated scales. Social network data were used to generate a set of variables that included the number of incoming and outgoing relational ties for each individual. Demographic variables were also included.

#### Independent Variable

##### Educator Wellbeing

This scale was based on the satisfaction with life scale (Diener et al., 1985) and the flourishing scale (Diener et al., 2010). Items from these scales were adapted for working in an educational context. For example, “the conditions of my life are excellent” was adapted to “my working conditions are excellent,” and the original item “my social relationships are supportive and rewarding” was replaced with two items “my relationships with work colleagues are supportive and rewarding” and “my relationships with students are rewarding.” Items were reviewed by educators and educational experts. The resulting educator wellbeing scale comprised 15 items where respondents rated their agreement using a 6-point agreement scale ranging from 1 (*strongly disagree*) to 6 (*strongly agree*). Data collected during this study were used to check the factor structure and validate the scale. We used a random sample-splitting method to perform exploratory factor analysis (EFA) followed by confirmatory factor analysis (CFA) as this is a practical method recommended to validate results (Woods and Edwards, 2007). EFA was conducted with a sample size of *N* = 213, using oblique rotation, principal axis factor extraction, and factors were retained according to Horn’s parallel analysis (Hayton et al., 2004). This yielded a three-factor solution, which was validated using CFA on the other half of the randomly split sample. In order to improve the CFA model fit, the factor loadings and modification indices were inspected, and two items were removed, providing a final model fit that was acceptable (*χ*^2^/df, *p* = 0.156; SRMR = 0.0673; gamma hat = 0.92) and scale reliability (Cronbach’s alpha 0.65–0.84). The final three factor solution from the CFA (see Table 2) was used in all subsequent analyses. The three factors were: satisfaction (items related to educators’ feelings of satisfaction with work), contribution (items related to meaning and contributing to the wellbeing of others), and support (items related to supportive and respectful relationships).

**Table 2:** Confirmatory factor analysis for the educator wellbeing scale.

		Factor loadings		
		F1	F2	F3
**Factor 1: Satisfaction**				
11	In most ways my educational work is close to my ideal.	0.743		
12	My working conditions are excellent.	0.712		
13	I am satisfied with my work.	0.811		
14	So far I have the important things I want from my work.	0.802		
**Factor 2: Contribution**				
1	My work as an educator is meaningful.		0.627	
3	My relationships with students are rewarding.		0.623	
4	I am engaged in my daily professional activities.		0.686	
5	I actively contribute to the well-being of my colleagues.		0.685	
6	I actively contribute to the well-being of students.		0.657	
**Factor 3: Support**				
2	My relationships with work colleagues are supportive.			0.682
7	I feel I have mastered the professional activities that are important to me.			0.425
8	I feel like I can be myself at work.			0.583
10	I feel respected by my colleagues.			0.707
	Cronbach’s Alpha	0.84	0.788	0.651
	MacDonald Omega	0.851	0.791	0.688

#### Dependent Variables

##### Network Intentionality

This scale measured educators’ intentions to create, broker, maintain, and assess their relationships with colleagues using four dimensions: (1) actively seeking relationships, (2) the belief in having the right relationships, (3) assessing relationships, and (4) liking to connect (Moolenaar et al., 2014). The wording of items was adapted to be specific to school contexts, for example, references to “the organization” in original items were replaced with “my school.” The scale comprised 14 items and used a 6-point agreement scale ranging from 1 (*strongly disagree*) to 6 (*strongly agree*). The scale has been validated in previous studies and shown to have on overall reliability of *α* = 0.84 (Moolenaar et al., 2014) and also showed good reliability for our data (Cronbach’s alpha = 0.89).

##### School Conditions

The measures of school conditions comprised two scales: trusting relationships and resources for collaboration. The *trusting relationships* scale measured educators’ perceptions of respect, trust, and confidence in other educators. The scale is based on the teacher-teacher trust scale by Bryk and Schneider (2002) with two items adapted, for example, “To what extent do you feel respected by other teachers?” was changed to an agreement scale for “I feel respected by other teachers.” One extra item was added as: “I have confidence in the expertise of other teachers.” The final scale comprised seven items and used a 6-point agreement scale ranging from 1 (*strongly disagree*) to 6 (*strongly agree*). The scale has been validated in previous studies and shown to have a reliability of *α* = 0.94 (Liou et al., 2019) and also showed good reliability for our data (Cronbach’s alpha = 0.90). The *resources for collaboration* scale measured educators’ perceptions of the opportunities to collaborate, including time and communication systems that enabled collaboration. The scale comprised five items and used a 6-point agreement scale ranging from 1 (*strongly disagree*) to 6 (*strongly agree*) and showed good scale reliability for our data (Cronbach’s alpha = 0.87).

##### Network Data

We collected data about educators’ social networks for advice and close relationships. A network roster (a list of all staff in the schools in each CoL) was presented to educators, and they indicated with whom they interacted. For the advice network, respondents indicated the frequency with which they turn to each of their colleagues in the CoL for advice about curriculum (daily, weekly, monthly, termly, or yearly). We collected binary data about close relationships, where respondents indicated individuals with whom they have a close relationship. The participants’ responses provided directed network data, indicating the direction of a relationship between pairs of individuals, which may not necessarily be reciprocated. For example, person A may seek advice from person B, but person B does not seek advice from person A. Network data were processed to provide a number of measures for individual educators (see Data Analysis section for more information).

### Control Variables: Demographics

The demographic information collected were educators’ formal roles within the school, gender, and the number of years they had worked as an educator.

### Data Analysis

Data were analyzed in two stages. Firstly, social network data were analyzed to produce a number of network measures for individuals based on their position in the network, membership of informal communities within the network, and the wellbeing of the individuals to whom they were connected in the network. Secondly, correlations and a series of hierarchical regressions were conducted to examine the associations between educator wellbeing and the independent and control variables.

#### Missing Values

All scale data were inspected for missing values. For cases where individuals had <10% missing data, the missing values were imputed; for cases with >10% missing data, the data were excluded—the value of 10% being a commonly accepted cutoff point in educational studies (Brown, 2016). Missing data were imputed using the expectation maximization method (Dempster et al., 1977), following confirmation that data were Missing Completely at Random (MCAR).

#### Social Network Data

##### Network Interactions

Centrality is the term used to encompass a set of measures that provide information about an individual’s position in a social network. The simplest measure of centrality is degree, which is simply the number of relational ties that an individual has (Borgatti et al., 2018). Outdegree indicates the number of people that a person has nominated in a particular network, for example, an outdegree of five for advice indicates that person seeks advice from five others. Indegree is the number of times an individual has been nominated by others in a network. We used the software UCINET (Borgatti et al., 2002) to calculate the outdegree and indegree for each individual for advice (monthly or more frequent) and close relationship. We used UCINET to generate normalized values, where normalization divides the degree value by the maximum possible degree to generate a value between zero and one.

##### Informal Network Community Membership

The social network package igraph for R (Csardi and Nepusz, 2006) was used to run community detection algorithms to identify the informal communities present within the networks. The infomap algorithm uses a random walk approach (Rosvall and Bergstrom, 2008) where a random starting point in the network is chosen and a path is traced as ties are randomly traversed from one node (person) to the next. For each tie, the probability that a random walk will traverse that tie is calculated, and groups of nodes (people) for which the random walk path is likely to remain within that group are identified as a community. The infomap algorithm was used as this is compatible with directed networks (those that specify whether ties are incoming or outgoing, rather than simply the presence of a tie) and demonstrates high-quality community detection when validated using known community structures (Singh and Garg, 2020).

##### Network Exposure Index

**Figure 2:**
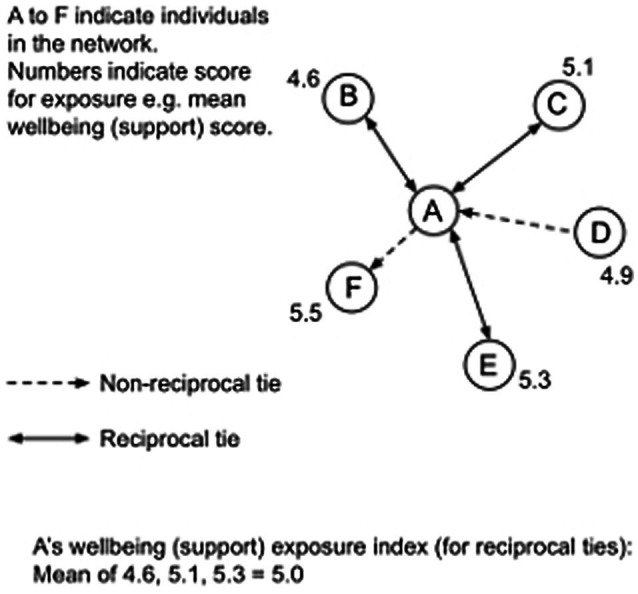
Exposure index calculation example.

An “exposure index” was calculated in order to examine the influence of an individual’s peers. This technique involves calculating the mean value of a score (such as a psychometric scale) for the group of people with whom an individual is directly connected in a network. An exposure index has been used in previous research to demonstrate how leaders’ perceptions are influenced by the beliefs of their peers (Liou and Daly, 2020). In this study, UCINET was used to generate matrices that contained only reciprocal ties for close relationships (i.e., ties between two individuals who had both nominated each other as someone with whom they have a close relationship). Each individual’s scores for each of the three wellbeing factors (satisfaction, contribution, and support) were combined with the matrix of reciprocal ties for close relationships to calculate the exposure index for each individual for each wellbeing factor (see Figure 2 for an example).

#### Correlation and Hierarchical Regression

Pearson’s correlations and a series of hierarchical regressions were conducted using the demographic data, mean scores for each of the scales (network intentionality, trusting relationships, and resources for collaboration), and the network measures (indegree, outdegree, community membership, and exposure index) to examine their associations with educator wellbeing. The hierarchical blockwise regressions were conducted to determine the variance in each wellbeing factor explained by the individual, relational, and organizational level measures that were over and above the variance explained by the demographic information. For each of the three wellbeing factors (satisfaction, contribution, and support), a series of five regressions were conducted. Each regression comprised the demographic variables, for which a block was then added for: (1) network intentionality, (2) network interactions, (3) informal network community membership, (4) network exposure index, and (5) school conditions.

## Results

### Descriptive Statistics

Our sample comprised 469 educators that completed the questionnaire (from a total of 559 educators in 12 schools, an 84% response rate). Demographic information for the sample is shown in Table 1. The educators had a mean of 15 years in the profession, and nearly three quarters of educators were female. Educators were employed in the primary sector (42% in primary and intermediate schools), the secondary sector (50%), and in composite schools that span primary and secondary years (7.7% of educators). In terms of their formal role, teachers with no leadership responsibility formed the largest group, followed by middle leaders (such as heads of department or year groups), with teacher aides and senior leaders each comprising less than 9% of the sample.

The **educator wellbeing scale** comprised three factors: satisfaction, contribution, and support. The mean scores for each of the three wellbeing scale factors were 4.62 (SD = 0.74) for satisfaction, 5.42 (SD = 0.50) for contribution, and 4.94 (SD = 0.60) for support. The **network intentionality scale** comprised four factors: seeking (actively seeking relationships), beliefs (the belief in having the right relationships), assessment (assessing relationships), and connect (liking to connect). The means of these factors ranged from 3.87 (SD = 1.17, for assessment) to 5.22 (SD = 0.65, for beliefs). Finally, the measures of school conditions are given by the **trusting relationships scale** (*M* = 4.95, SD = 0.66) and **the resources for collaboration scale** (*M* = 4.61, SD = 0.72). All variables were measured on a 6-point scale.

**Figure 3:**
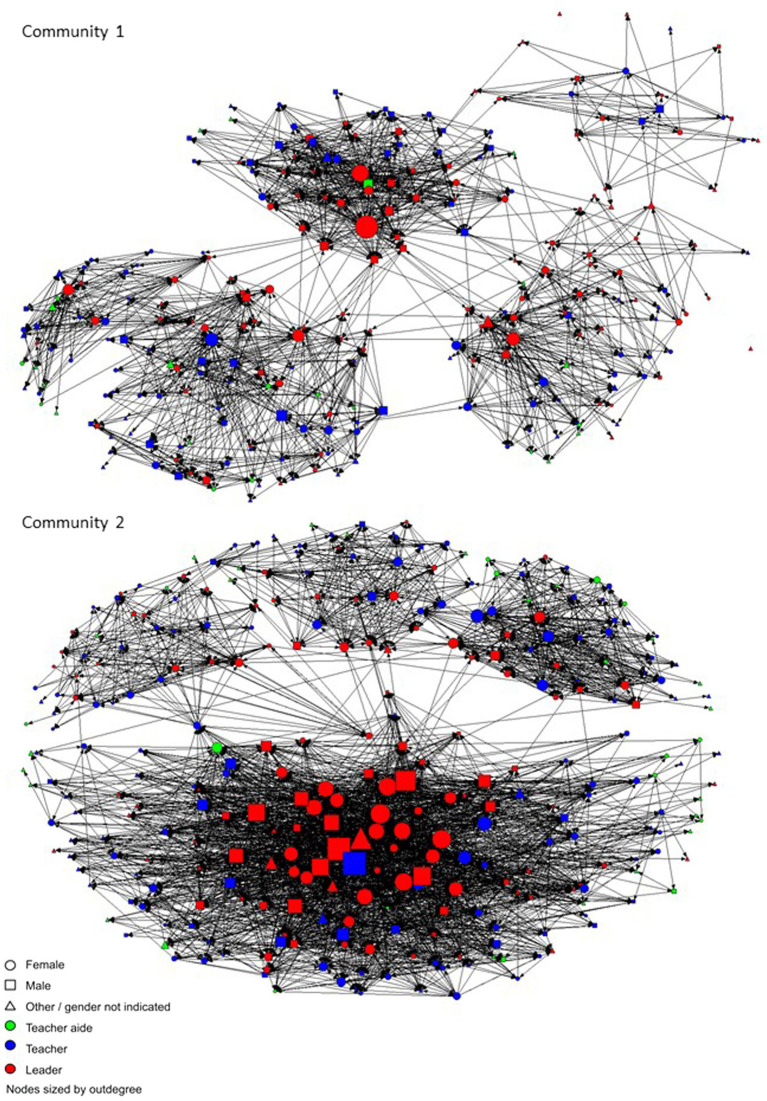
Advice ties (monthly or more frequent).

**Figure 4:**
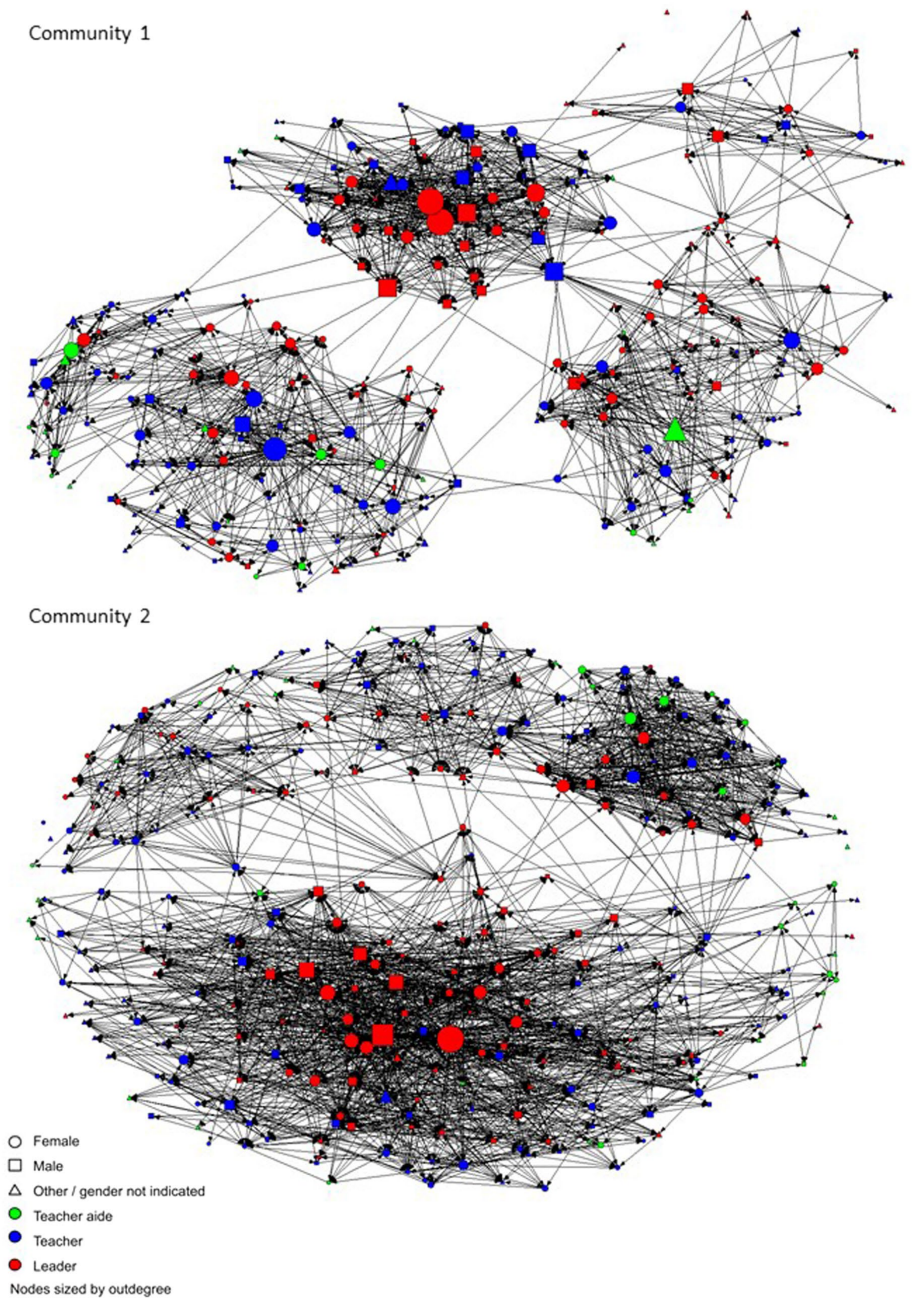
Close relationship ties.

The social network data are portrayed graphically in the sociograms in Figures 3, 4. Figure 3 shows each of the two Communities of Learning, with each educator represented by a shape, and the lines between them representing monthly or more frequent advice ties, where respondents indicated who they turn to for advice about the school curriculum. Figure 4 shows the close relationship ties, a binary measure, where respondents indicated individuals with whom they have a close relationship. The social network data were used to calculate the centrality measures of outdegree and indegree for each individual for advice (monthly or more frequent) and close relationships. Outdegree indicates the number of people that a person has nominated in a particular network, for example, an outdegree of five for advice indicates that person seeks advice from five others. Indegree is then the number of times an individual has been nominated by others in that network. The descriptive data in Table 1 show that for close relationships outdegree the mean number of ties were 9.6 (SD = 10.8), and for indegree *M* = 8.49 (SD = 5.4). For advice seeking ties, the outdegree *M* = 12.1 (SD = 14.4) and for indegree *M* = 11.1 (SD = 8.9). The indegree and outdegree data were normalized to convert the number of ties to a value between zero and one for use in the Pearson’s correlations and hierarchical linear regressions.

**Figure 5:**
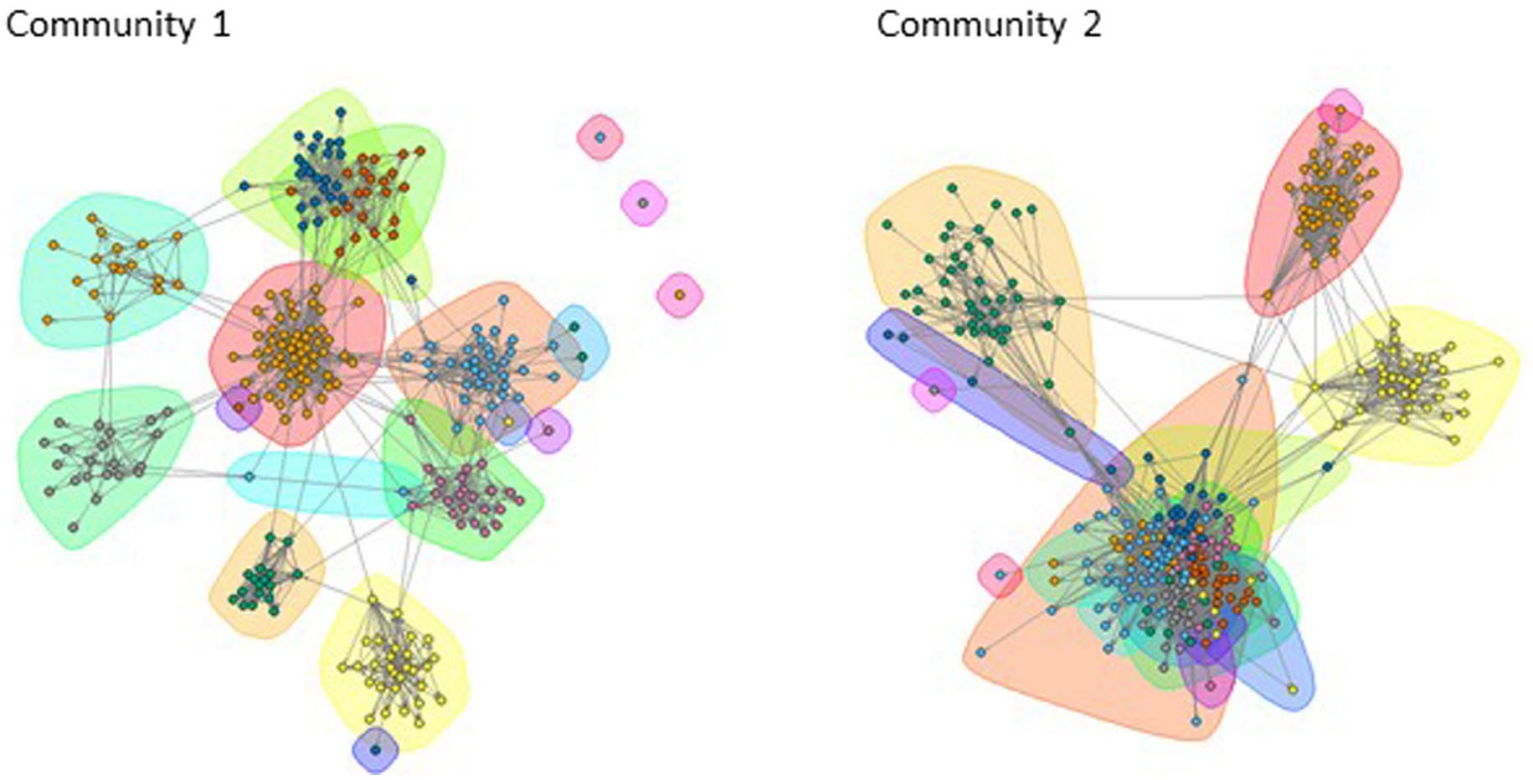
Infomap communities identified for the 12 schools.

In order to detect the informal communities (groups of individuals that are most likely to interact with each other), the infomap algorithm was applied to the monthly advice seeking networks for each Community of Learning. Each colored area in the maps in Figure 5 shows a community (note that an individual only belongs to one community—any overlapping colored areas are a function of the layout of the nodes representing the educators). A total of 35 communities were detected across the two Communities of Learning, 22 of these communities had 4 or more members (20 communities ≥10 people), and membership of these communities was included in the regression analyses.

### Relationships With Educator Wellbeing

The results of Pearson’s correlations show that all continuous variables, apart from educator’s monthly advice outdegree, were significantly correlated with at least one of the three dimensions of educator wellbeing (see Table 3). These variables were then included in a series of hierarchical linear regressions, with each of the three educator wellbeing dimensions (satisfaction, contribution, and support) as the dependent variable (Table 4). The demographic variables were entered as one block, and a series of analyses were performed with blocks added to explore how individual, relational, and organizational factors contributed to educator wellbeing, over and above the role of demographics. We conducted five regressions across the three factors:

- individual factors: (1) network intentionality.
- relational factors: (2) network interactions, (3) informal network community membership, and (4) network exposure index.
- organizational factors: (5) school conditions.

**Table 3:** Correlations between the three wellbeing dimensions and other variables.

	1	2	3	4	5	6	7	8	9	10	11	12	13	14	15	16
1. Wellbeing (satisfaction)	–															
2. Wellbeing (contribution)	0.433^**^	–														
3. Wellbeing (support)	0.643^**^	0.520^**^	–													
4. Years as an educator	0.199^**^	0.157^**^	0.194^**^	–												
5. Monthly advice outdegree (norm)	−0.040	0.077	0.006	−0.022	–											
6. Monthly advice indegree (norm)	−0.052	0.103^*^	0.035	0.219^**^	0.486^**^	–										
7. Close relationship outdegree (norm)	0.021	0.157^**^	0.159^**^	0.039	0.321^**^	0.372^**^	–									
8. Close relationship indegree (norm)	−0.032	0.107^*^	0.115^*^	0.231^**^	0.354^**^	0.685^**^	0.413^**^	–								
9. Close contacts (reciprocal) Wellbeing (sat)	0.299^**^	0.162^**^	0.239^**^	0.166^**^	0.006	−0.025	0.033	0.013	–							
10. Close contacts (reciprocal) Wellbeing (con)	0.129^*^	0.108^*^	0.133^*^	0.123^*^	0.050	0.049	0.061	0.099	0.533^**^	–						
11. Close contacts (reciprocal) Wellbeing (sup)	0.251^**^	0.106^*^	0.307^**^	0.180^**^	0.104^*^	0.055	0.155^**^	0.154^**^	0.668^**^	0.530^**^	–					
12. Network intentionality (seek)	0.194^**^	0.224^**^	0.258^**^	−0.120^*^	0.034	−0.049	0.122^*^	0.031	0.096	0.012	0.090	–				
13. Network intentionality (beliefs)	0.131^**^	0.270^**^	0.169^**^	−0.166^**^	0.023	−0.035	0.067	0.001	0.050	−0.066	0.098	0.492^**^	–			
14. Network intentionality (assess)	0.122^*^	0.119^*^	0.140^**^	−0.038	0.027	−0.091	0.070	−0.004	0.049	0.009	0.052	0.373^**^	0.230^**^	–		
5. Network intentionality (connect)	0.107^*^	0.262^**^	0.196^**^	0.006	0.069	0.065	0.131^**^	0.150^**^	0.041	0.002	0.063	0.486^**^	0.431^**^	0.497^**^	–	
16. Resources for collaboration	0.535^**^	0.313^**^	0.426^**^	0.136^**^	−0.040	0.003	−0.050	−0.035	0.253^**^	0.086	0.183^**^	0.203^**^	0.160^**^	0.015	0.105^*^	–
17. Trusting relationships	0.423^**^	0.320^**^	0.537^**^	0.020	−0.053	−0.032	0.046	0.029	0.222^**^	0.087	0.262^**^	0.223^**^	0.267^**^	−0.034	0.108^*^	0.542^**^

** Correlation is significant at the 0.01 level (two tailed).

* Correlation is significant at the 0.05 level (two tailed).

**Table 4:** Hierarchical lineaer regressions: Educator wellbeing regressed into (1) network intentionality, (2) network interactions, (3) network community membership, (4) network exposure index, and (5) school conditions.

	Wellbeing								
	Satisfaction			Contribution			Support		
	Beta		Beta		Beta	
**(1) Demographics**									
Female	0.027		0.090		0.035	
Teacher	−0.209		−0.200		−0.119	
Leader	−0.231 *		−0.041		−0.107	
Years as an educator	0.229 ***		0.156 **		0.222 ***	
% variance explained by this block		3.2%		5.3%		2.7%
**Network intentionality**									
Network intentionality (seek)	0.179 **		0.114		0.225 ***	
Network intentionality (beliefs)	0.082		0.215 ***		0.070	
Network intentionality (assess)	0.083		−0.033		0.023	
Network intentionality (connect)	−0.056		0.102		0.036	
% variance explained by this block		**4.9%**		**15.9%**		**7.9%**
**(2) Demographics**									
Female	0.020		0.109		0.013	
Teacher	−0.126		−0.133		−0.078	
Leader	−0.102		0.033		−0.042	
Years as an educator	0.220 ***		0.121 *		0.183 ***	
% variance explained by this block		3.4%		5.3%		2.9%
**Network interactions**									
Monthly advice outdegree (norm)	0.016		0.039		−0.019	
Monthly advice indegree (norm)	−0.094		−0.038		−0.135	
Close relationship outdegree (norm)	0.071		0.149 **		0.157 **	
Close relationship indegree (norm)	−0.066		−0.032		0.088	
% of variance explained by this block		**0.3%**		**1.2%**		**2.0%**
**(3) Demographics**									
Female	0.038		0.112 *		0.053	
Teacher	−0.128		−0.108		−0.077	
Leader	−0.155		0.050		−0.042	
Years as an educator	0.193 ***		0.095		0.163 **	
% variance explained by this block		3.4%		5.3%		2.9%
**Informal network community membership**									
Community 2	−0.083		−0.092		−0.265 *	
Community 3	−0.178		−0.204		−0.350 **	
Community 4	−0.020		−0.142		−0.272 *	
Community 11	−0.011		−0.120		−0.163 *	
Community 22	−0.074		−0.146		−0.218 *	
17 other communities with no significant association to wellbeing scores						
% variance explained by this block		**4.5%**		**1.9%**		**5.5%**
**(4) Demographics**									
Female	0.046		0.165 **		0.060	
Teacher	−0.104		−0.191		−0.026	
Leader	−0.095		−0.029		−0.001	
Years as an educator	0.126 *		0.090		0.121 *	
% variance explained by this block		2.7%		6.5%		2.4%
**Exposure index**									
Close contacts (reciprocal) Wellbeing (sat)	0.244 ***		0.132		0.085	
Close contacts (reciprocal) Wellbeing (con)	−0.100		−0.055		−0.098	
Close contacts (reciprocal) Wellbeing (sup)	0.127		0.044		0.278 ***	
% variance explained by this block		**7.8%**		**1.1%**		**7.7%**
**(5) Demographics**									
Female	0.037		0.114 *		0.061	
Teacher	−0.157		−0.100		−0.127	
Leader	−0.173		0.074		−0.099	
Years as an educator	0.137 **		0.076		0.149 ***	
% variance explained by this block		3.4%		4.5%		2.9%
**School conditions**									
Professional relationships	0.206 ***		0.244 ***		0.448 ***	
Resources for collaboration	0.420 ***		0.175 **		0.157 **	
% variance explained by this block		**31.1%**		**13.3%**		**30.2%**

Percentages in bold indicate the variance in each wellbeing factor explained by the individual, relational, and organizational level measures that were over and above the variance explained by the demographic variables. *** p < 0.001; ** p < 0.01; and * p < 0.05.

### Individual Level Factors

The demographic variables were associated with 3 to 6% variation in wellbeing. The number of years that an individual had been working as an educator was statistically significantly and positively associated with educator wellbeing for a number of regressions, indicating support for hypothesis 2a. However, there was little support for hypothesis 2b, that having a leadership role is negatively associated with wellbeing, as being a leader was generally not significantly associated with wellbeing when other variables were included in the regression. The only exception was that being a leader was significantly and negatively associated with wellbeing (satisfaction) levels when the regression included network intentionality measures. There was no support for hypothesis 2c that women will have lower wellbeing, as there were three instances where being female was significantly and positive associated with wellbeing (contribution) when the regression included variables related to informal communities, exposure index, and school climate.

There was support for hypothesis 1, that an individual’s network intentionality is positively associated with wellbeing, with network intentionality explaining 4.9 to 15.9% for variation in wellbeing above that explained by demographic variables. The largest variation explained was for the contribution factor of wellbeing. However, only two of the four network intentionality factors were significantly associated with wellbeing: seeking (actively seeking relationships) and beliefs (the belief in having the right relationships).

### Relational Level Factors

Educators network interactions showed little association with educator wellbeing. The close relationships outdegree was significantly and positively associated with wellbeing; therefore, the greater the number of close contacts a person names, the higher their wellbeing. However, close relationships indegree, and advice indegree and outdegree were not significantly associated with wellbeing, showing that overall, there was little support for hypothesis 3. In addition, the indegree and outdegree variables only explained between 0.3 and 2% of the variation in wellbeing, so in practical terms, this effect is trivial.

There was an association between network exposure index and wellbeing (satisfaction and support), explaining 7.7 to 7.8% variation in wellbeing above that explained by the demographic variables, showing some support for hypothesis 4a. The wellbeing (satisfaction) of people to whom an individual was connected through reciprocated close relationships was significantly and positively associated with that individual’s wellbeing (satisfaction). The same was also true for wellbeing (support); the higher that people rated wellbeing (support), the higher the rating of wellbeing (support) for the people they were connected to through reciprocated close relationships.

Membership of some of the informal network communities did show a significant association with wellbeing. From the 12 schools, we detected 22 communities with membership ≥4 educators (20 communities ≥10 people) and included these in the regression as dummy variables. Membership of five communities was negatively associated with the support factor of wellbeing. T-tests revealed significantly lower levels of wellbeing (support) for educators in these five communities (*M* = 4.74, SD = 0.59) compared to the other communities (*M* = 5.02, SD = 0.60), *t*(429) = −4.43, *p* = < 0.001. However, there were no significant differences between the levels of wellbeing (satisfaction) for educators in these five communities (*M* = 4.54, SD = 0.46) compared to the other communities (*M* = 4.66, SD = 0.74), *t*(429) = −1.60, *p* = 0.110. There were 158 members across the five communities identified, and 415 educators included in the regression for communities (listwise deletion), so this corresponds to 38% of educators for whom their informal network is negatively associated with wellbeing. However, these effects are small, with the model explaining 7.7% of the variation in wellbeing (support). Therefore, hypothesis 4b is partially supported, as membership of some informal communities was negatively associated with wellbeing; however, we did not find any evidence that belonging to an informal community was positively associated with wellbeing.

### Organizational Level Factors

Educators’ perceptions of trusting relationships and resources for collaboration were both significantly and positively associated with all three wellbeing factors. This demonstrates good support for hypothesis 5. The two scales together explained a large amount of the variation in wellbeing above that explained by the demographic variables (satisfaction: 31%, contribution: 13%, and support: 30%).

## Discussion

Our study adds to the literature on educator wellbeing by exploring the relative contributions of individual, relational, and organizational factors to educator wellbeing. Findings showed that school conditions explained the largest variation in educator wellbeing, greater than that explained by any of the individual or relational factors. While good social support has been shown to help reduce teacher stress (Kyriacou, 2001), little research has used social network analysis to explore the links between educators’ relational ties and wellbeing. Our findings based on social network data indicate that although the number of relational ties is positively and significantly associated with wellbeing, they explain little variation in wellbeing. In this study, the largest variation in wellbeing was explained by the school conditions of trust and collaboration, underscoring the importance of systems thinking in positive psychology research in order to explore the role of organizational conditions in influencing wellbeing (Kern et al., 2020). The results of our series of hierarchical linear regressions are discussed to determine whether our hypotheses were supported, partially supported, or not supported (see Table 5 for details), and their relation to previous research.

**Table 5:** Hypotheses.

Hypothesis	Supported, partially supported, or unsupported
Hypothesis 1: An individual’s level of network intentionality will be positively associated with wellbeing.	Partially supported Two of the four factors of network intentionality were significantly associated with wellbeing. Explained the most variation in wellbeing (contribution) at 16%
Hypothesis 2: Demographic variables will be associated with wellbeing: Years of experience will be positively associated with wellbeing. Having a leadership role will be negatively associated with wellbeing. Being female will be negatively associated with wellbeing.	Partially supported Years of experience positively associated with wellbeing in all regressions. Leadership position negatively associated with wellbeing (satisfaction) in one regression. But, being female positively associated with wellbeing (contribution) in three regressions.
Hypothesis 3: The number of relational ties an educator has will be positively associated with their wellbeing.	Supported However, the variation in wellbeing explained is very small (< 2%)
Hypothesis 4: The positive resources available to educators through their connections (such as high wellbeing or support provided) will be positively associated with wellbeing: The wellbeing of the people an individual has relational ties with will be positively associated with that individual’s wellbeing. The informal community to which an individual belongs will be associated with wellbeing (either positively or negatively depending on the nature of the community).	Supported Exposure index positively associated with wellbeing. Five communities with significantly lower levels of wellbeing were negatively associated with educator wellbeing.
Hypothesis 5: An individual’s perception of trusting and collaborative school conditions will be positively associated with the individual’s wellbeing.	Supported Explained the most variation in wellbeing overall (31% for satisfaction, 13% for contribution, and 30% for support)

### Individual Level Factors (Hypotheses 1 and 2)

As predicted in hypothesis 1, educators’ network intentionality was positively associated with wellbeing. After taking into account demographic variables, network intentionality explained 5–16% additional variance in wellbeing. Two of the four factors of the network intentionality scale were significantly associated with wellbeing: seeking (actively seeking relationships) and beliefs (the belief in having the right relationships).

Educators’ disposition toward seeking relationships may be associated with higher wellbeing as social support plays a role in alleviating negative states, such as stress, and promoting better wellbeing. Previous studies have shown that high levels of network intentionality are associated with more outgoing relational activity (Moolenaar et al., 2014), and in our study, network intentionality (seeking) was significantly associated with close relationships outdegree (0.122, *p* < 0.05). Seeking assistance from colleagues and connecting with others are positive coping strategies that help teachers to adapt to new situations and build their self-efficacy (Sharplin et al., 2011). Social support has also been shown to be significantly associated with decreases in teacher stress and burnout, and increases in job satisfaction (Kyriacou, 2001; Kinman et al., 2011). Social support also influences the relationship between individual resources and teacher wellbeing, for example, mediating the link between teachers’ emotional intelligence and burnout (Mérida-López and Extremera, 2017), and moderating the relationship between teachers’ psychological capital and work-related wellbeing (Li and Zhang, 2019). However, as discussed in the section on relational factors, although the quantity of outgoing close relationship ties was significantly associated with wellbeing, the number of relational ties within schools explained very little variation in educator wellbeing (0.3 to 2.0%). This suggests that there are other mechanisms by which educators’ disposition toward seeking relationships is associated with wellbeing. One reason may be that while our study examined the relational ties within schools, social support outside of school is also an important influence on educator wellbeing (Price and McCallum, 2015).

Social reciprocity may explain the association between the wellbeing factor contribution (which included individuals’ tendency to help others) and educators’ beliefs that having the right relationships can positively influence happiness and performance at work. The norm of social reciprocity includes positive reciprocity, whereby people help others who have helped them in the past, and negative reciprocity, where individuals retaliate against others that have negatively impacted them (Perugini et al., 2003). People who have a tendency for positive reciprocation (rather than negative) are more likely to notice and react to positive interpersonal events (Perugini et al., 2003). In our study, the educators with beliefs that relationships can positively influence happiness and performance at work may have been more likely to demonstrate positive reciprocal behavior through contributing to the wellbeing of others.

Hypothesis 2 was partially supported, as the demographics varied in their association with wellbeing. In our study, the number of years as an educator was linked with higher wellbeing in all regression models, supporting hypothesis 2a, and consistent with other studies that the longer teachers remain in the profession, the greater their levels of positive affect and the less burnout they experience (McCarthy et al., 2009; Gloria et al., 2013). One possible explanation for this is that teachers who find the profession particularly challenging will leave. Up to 50% of teachers leave in the first 5 years, with many citing high workload and a lack of support as contributing to their decision to leave (Australian Institute for Teaching and School Leadership, 2016). The longer that teachers remain in the profession, the greater their self-efficacy, which in turn is associated with higher levels of job satisfaction (Klassen and Chiu, 2010). Hypothesis 2c, that women will have lower wellbeing levels than others, was not supported, which is inconsistent with other literature showing women have lower levels of employee wellbeing (Ravenswood et al., 2017) and female teachers experience higher levels of stress (Klassen and Chiu, 2010). However, in our study, being female was significantly and positively associated with the contribution factor of wellbeing, which encompassed perceptions of meaningful relationships with students and colleagues, and contributing to the wellbeing of others. This may be explained due to women being more likely than men to engage in helping and supportive relational behaviors and that women are more likely to expect those behaviors to positively enhance their mood (Sprecher et al., 2007). Finally, there was little support for hypothesis 2b, that having a leadership role will be negatively associated with wellbeing, which may be explained by the greater degree of autonomy that leaders experience offsetting the greater demands placed upon them. While previous studies report that principals experience high levels of stress (Wylie and MacDonald, 2020), and teachers associate middle leadership roles with higher workload and pressure (Cameron et al., 2007), these disadvantages may be offset as leaders have greater control over their work which can reduce their experience of stress (Marmot, 2004).

### Relational Factors (Hypotheses 3 and 4)

Our findings show that the quantity of relational ties predicts very little variation in wellbeing, and we highlight the importance of considering the quality of relational interactions. Although the number of close relationships that educators reported were significantly associated with wellbeing, supporting hypothesis 3, educators network interactions (close relationships and advice seeking) only explained 0.3–2.0% variation in wellbeing, the least variation explained by any of the factors we considered. One reason that the quantity of relational ties explained the least variation in wellbeing in our study may be because the quality of interactions has more influence than the quantity. For example, Park (2004) notes that the quality of social interactions is more important than the quantity of interactions in developing young peoples’ subjective wellbeing. The quality of relationships has also been shown to be a stronger predictor of self-reported health than the quantity of relationships (Fiorillo and Sabatini, 2011). We reach a similar conclusion in our study—that in terms of explaining educator wellbeing the quality of interactions is more important than the quantity. As discussed in the section on organizational level factors, our findings show that educators’ perceptions of trusting and collaborative school conditions (measures that indicate quality relationships) explain the most variation in wellbeing—far more than is explained by the quantity of educators’ relational ties.

Wellbeing was associated with both informal network community membership and network exposure index, supporting hypotheses 4a and 4b. We propose this is due to these measures capturing the quality of interactions in an educator’s immediate network that are influencing their wellbeing. Previous studies have demonstrated how the behaviors and attitudes of connections within a social network can influence an individual’s wellbeing. For example, social proximity to people who perform prosocial acts has been associated with increases in wellbeing (Chancellor et al., 2018). The influence of the social network could be direct or indirect. For example, Kim et al. (2017) found that early career teachers were more likely to suffer burnout when they directly interacted with teachers who were burnt out (they termed this social network exposure). Also, being indirectly exposed to burnout due to a greater mean level of burnout in their school (they termed this organizational exposure) led to higher levels of burnout (Kim et al., 2017). Our findings demonstrate a similar phenomenon with social network exposure to wellbeing—the greater the wellbeing of an educator’s direct connections, the greater their level of wellbeing. We also found that five of the 22 informal network communities had significantly lower levels of wellbeing (support) and were associated with lower wellbeing (support) levels. This finding is similar to the effect of organizational exposure found by Kim et al. (2017) where teachers’ levels of burnout were likely to be higher when the mean level of burnout in a school was higher. This effect is less focused on an educator’s direct connections and instead refers to the general level of resources in a school. One possible explanation for this indirect effect could be the “witnessing effect,” where emotional expressions observed by third party witnesses can influence the way in which the witness intends to interact with the people they observed (Algoe et al., 2020). When a witness observed expressions of gratitude between two people, they were more likely to express a desire to be helpful and affiliative toward those people (Algoe et al., 2020). We suggest that the witnessing effect could explain the lower levels of wellbeing (support) in the five “low support” communities—if educators do not observe supportive and respectful relationships between others then, they are less likely to behave in supportive and respectful ways to others. However, further research would be needed to confirm this hypothesis.

When interpreting these results caution should be exercised as reverse causality may apply. It may be that the social circumstances that have been observed, for example membership of a certain informal community, may not be influencing wellbeing, but instead are as a result of an individual’s level of wellbeing (Helliwell and Putnam, 2005). Rather than social influence occurring, where an individual’s social circumstances influence their wellbeing, it may be that social selection is occurring, where individuals “form social relationships on the basis of certain characteristics they possess” (Robins et al., 2001, p. 1), in this case their level of wellbeing. In observational social network, studies homophily (when social network ties are formed based on matching individual traits) and social influence are often confounded (Shalizi and Thomas, 2011). However, it is likely that social selection and social influence occur simultaneously (Robins et al., 2001). For example, Schulte et al. (2012) have identified that within workplaces social network ties and employees’ perceptions of psychological safety co-evolve over time, and it may be that a similar process occurs in terms of educator wellbeing. Further research would be needed to determine causality, including the relative contributions of social influence and social selection to the phenomenon of educator wellbeing.

### Organizational Level Factors (Hypothesis 5)

In our study, wellbeing was most strongly associated with trusting and collaborative school conditions. The links between wellbeing and trust may be explained as trust leads to prosocial behavior and more frequent social interactions. Our findings add to the literature that generalized trust, trust in work colleagues, and collaborative environments are associated with subjective wellbeing and job satisfaction (Helliwell and Wang, 2010; Schneider et al., 2013; Di Fabio, 2017). In particular, we add to the literature that educator’s trust in their colleagues is associated with higher levels of wellbeing (Bjorklund et al., 2021) in addition to reducing their levels of burnout, anxiety, and depression (Van Maele and Van Houtte, 2015; Yin et al., 2018; Huang et al., 2019). One reason for this may be because trust engenders prosocial behaviors, such as cooperation and altruism, that enhance wellbeing (Kramer, 1999). Another reason for the association between trust and wellbeing is due to increased social interaction. Higher levels of teacher-teacher trust have been linked to more frequent network connections with colleagues (Liou and Daly, 2014), and more frequent social interactions are associated with greater positive affect (Watson et al., 1992; Sandstrom and Dunn, 2014).

Our findings show collaboration was strongly associated with wellbeing, which may be explained due to collaboration being linked to learning (Webb et al., 2009; Bjorklund et al., 2021), meaningful work (Shirley et al., 2020), and teacher self-efficacy (Leithwood., 2006; Voelkel and Chrispeels, 2017). Meaning, learning, growth, and accomplishment are dimensions of several models of wellbeing (Ryff, 1989; Seligman., 2011; Huppert and So, 2013; Mental Health Foundation of New Zealand, 2015), so if these dimensions are increased due to collaboration this would have an overall positive effect on wellbeing. Previous studies have identified collaboration as a key element in cultures that promote teacher wellbeing and reduce the impact of negative aspects of the job (Roffey, 2012; Paterson and Grantham, 2016; Wigford and Higgins, 2019). Well managed collaboration in schools, where educators feel included in decision making, can contribute to meaningful work that supports educators to improve their practice, leading to greater job satisfaction and wellbeing (Shirley et al., 2020). Collaboration is also positively associated with trusting relationships between school staff (Jäppinen et al., 2016), highlighting the links between trust and collaboration, and suggesting another reason why our findings show that both trust and collaboration are associated with wellbeing.

These findings highlight that wellbeing is more strongly associated with the quality of relationships, rather than the quantity. Collegial relationships that support, connect, and encourage positive emotions are essential for enabling educators to flourish (Acton and Glasgow, 2015). While frequent social interactions, even with weak ties, have been associated with greater happiness (Sandstrom and Dunn, 2014), the quality of interactions, rather than quantity, is a stronger predictor of health and wellbeing (Park, 2004; Fiorillo and Sabatini, 2011). In our study, quality of interactions is evident when educators rate school conditions as trusting and collaborative, and this is more strongly associated with educator wellbeing than their number of relational ties in their school network.

### Practical Implications

Based on our findings, we suggest two main practical implications for schools: (1) the importance of creating climates of trust and collaboration, and (2) the need for an awareness of wellbeing contagion.

Our findings showed that collaborative and trusting school conditions explained the most variation in educator wellbeing. However, the least variation in wellbeing was explained by the number of network interactions that educators reported (although these measures did not include a rating of the quality of the interaction). Therefore, a focus on creating trusting and collaborative school conditions is likely to have a greater impact on educator wellbeing than simply encouraging educators to interact more with their colleagues without any consideration of the quality of those interactions. School leaders are pivotal in creating positive school climates which can support educators to flourish (Acton and Glasgow, 2015; Cherkowski and Walker, 2018), and therefore, school leaders can play a significant role in influencing educator wellbeing. Creating climates of trust may also encourage positive attitudes toward seeking advice, as trusting relationships allow teachers to share their concerns and ask for advice (Liou and Daly, 2014). This is important as our findings also showed that individual’s attitudes toward seeking relationships within school were positively associated with wellbeing.

In terms of creating climates of trust, the construct of the psychological contract suggests ways in which employers can influence employee trust. A psychological contract refers to “employees’ perceptions of what they owe to their employers and what their employers owe to them” (Robinson, 1996, p. 574). If employees perceive a breach of the psychological contract—for example, through a lack of promotion, training, or job security—then this leads to a decline in trust in their employer (Robinson, 1996). In terms of teaching, fulfilment of the psychological contract has been shown to reduce teacher burnout, through the perceived support from their school (Brown and Roloff, 2011). School leaders can fulfill the psychological contract with teachers through support provided when they value teachers’ contributions, appreciate extra effort, and consider teachers’ goals, values, and best interests when making decisions affecting teachers (Brown and Roloff, 2011).

Our findings indicate that wellbeing may be contagious—that the wellbeing of people that educators interact with can impact their wellbeing—either through their direct interactions, or *via* membership of an informal community. This is consistent with social contagion theory, which demonstrates that a number of behaviors and affective states (such as smoking, obesity, and happiness) are spread through social networks *via* interpersonal influence (Christakis and Fowler, 2013). Other studies show happiness is contagious for both adults and adolescents (Fowler and Christakis, 2008; Van Workum et al., 2013), burnout is contagious among teachers (Kim et al., 2017), and job satisfaction and prosocial behavior are contagious among work colleagues (Chancellor et al., 2017). In our study, network exposure—the wellbeing levels of an individual’s direct connections—was associated with educator wellbeing. Also, some informal network communities were associated with lower educator wellbeing levels, suggesting that when community members have lower wellbeing, they have lower resources to support each other. The implication for schools is that they could take advantage of this possible wellbeing contagion effect to support educator wellbeing. Identifying key players, a group of people that connect with all others in the network (Borgatti, 2006), and improving their wellbeing, or their capacity to support others’ wellbeing, may have positive impacts on other educators in the network. For example, some workplaces have used the concept of “wellbeing champions,” employees who support the wellbeing of their colleagues, as a way of improving staff wellbeing (Wickramasinghe, 2020).

Social contagion also explains how leaders can influence their followers (Chancellor et al., 2017), and effective leaders know how to manage that contagion (Ashkanasy and Dorris, 2017). In particular, the emotions, attitudes, and actions of leaders can influence the spread of negative emotions. For example, when leaders practice organizational justice (fairness, respect, and clear communication) their teams have lower rates of negative workplace gossip (Cheng et al., 2021). When leaders foster positive working relationships, and instill a sense of vision and pride in their teams, then negative events have less impact on team climate (Pirola-Merlo et al., 2002). In schools, leaders are instrumental in determining whether negative cultures develop, through what they focus on, or neglect to focus on, such as shared planning time for staff, and valuing long standing customs and positive stories about the school (Deal and Peterson, 2016). Therefore, school leaders have an important role to play in reducing negative wellbeing contagion.

### Limitations and Future Research

Limitations of this study included the narrow range of measures included. Other studies show there are many factors that can influence educator wellbeing at the individual, relational, and organizational level. For example, at the relational and organizational level, educator wellbeing is influenced by student behavior (Aldrup et al., 2018; Collie et al., 2020), leadership support and recognition (Yildirim, 2015; Janovská et al., 2016), professional development (Webb et al., 2009; Collie et al., 2020), emotional job demands (Yin et al., 2018), feedback (Bermejo-Toro et al., 2016), and input into decision making (Collie et al., 2020). At the individual level, educator wellbeing is related to educators’ coping strategies (Bermejo-Toro et al., 2016; Aulén et al., 2021), resilience (Arnup and Bowles, 2016), and self-efficacy (Klassen and Chiu, 2010; Yildirim, 2015; Bermejo-Toro et al., 2016; Huang et al., 2019). Future studies could include different measures at the individual, relational, and organizational level and assess their relative influences on educator wellbeing. For example, the Big Five and HEXACO personality measures have been shown to be strongly associated with wellbeing across hundreds of studies (Anglim et al., 2020), but it has also been shown that workplace relational culture explains variance in wellbeing above that predicted by personality traits (Di Fabio et al., 2016). Future research could include a measures of educators’ personality traits to ascertain their association to wellbeing relative to relational and organizational variables, such as those used in this study.

Another limitation is that this study is observational and cannot prove cause and effect. For example, we found that some informal communities were negatively associated with educators’ rating of the support factor of wellbeing, but we cannot determine if this is social influence or social selection. Further research could explore the mechanism by which educators choose their social ties, and the degree to which wellbeing is influenced by their interactions in the school network.

Another limitation of the methodological approach is the grouping used for the network. In order to fully describe the relations within the boundaries of a specific group, we only included interactions between educators within two communities of learning (CoLs). Therefore, the data do not include interactions with others outside the CoLs. In order to overcome this limitation, future research could use an ego network approach that allows for interactions outside the CoLs to be included. Additionally, due to the intensive nature of the data collection, only two CoLs were included in this study, and therefore, the findings cannot be generalized to all CoLs in New Zealand. Future research could include a larger number of CoLs that are representative of CoLs across New Zealand.

The network interactions recorded were the frequency of interactions for advice (daily, weekly, or monthly) and the number of close relationships that educators reported. The reporting of these network interactions was focused on the quantity of connections—the frequency or presence of a tie—without any evaluation of the quality of the interactions. In contrast, when educators responded to items related to school conditions, they rated the quality of interactions in terms of the degree of trust and respect present in the interactions, and the opportunities for collaboration that included learning and productive dialogue. The findings showed that school conditions (the quality of interactions) had a much greater association with educator wellbeing than network interactions (the quantity of interactions), and therefore, underscores the importance of the quality of interactions over the quantity. However, the scales we used to capture educators’ perceptions of trusting relationships asked about relationships in general and did not capture information to evaluate the quality of interactions at the dyadic level. Therefore, given the significant association between the quality of interactions and wellbeing, further network survey research could be designed to capture the quality of respondents’ interactions with others at the dyadic level.

## Conclusion

Our study underscores the importance of the quality of interactions within schools, as trusting and collaborative school conditions explained the most variation in wellbeing, and we suggest that creating positive and supportive school conditions could support educator wellbeing. Our findings also demonstrated the phenomenon of wellbeing contagion, indicating that all educators within schools, not only those in positions of leadership, have the potential to influence the wellbeing of others. In conclusion, in order to create the environment needed for educators to flourish, we suggest that school leaders commit to building trusting and collaborative school conditions, and that all educators consider how they can influence those around them.

## Data Availability Statement

The datasets presented in this article are not readily available because ethics approval did not allow for the sharing of data. Requests to access the datasets should be directed to rachel.cann@auckland.ac.nz.

## Ethics Statement

The studies involving human participants were reviewed and approved by University of Auckland Human Participants Ethics Committee. The participants provided their written informed consent to participate in this study.

## Author Contributions

RC conceptualized the study, carried out the analysis, and prepared the manuscript draft with advice and feedback throughout from CS, AD, JR, and Y-HL. The project the data are drawn from was led by CS and AD with data preparation and analysis by JR, Y-HL, and RC. All authors contributed to the article and approved the submitted version.

## Funding

The manuscript fee is provided by the University of Auckland, School of Education and Social Work PBRF Fund, reference FILE 40234. The research time of Y-HL is supported by the Ministry of Science and Technology, R.O.C (MOST 108-2410-H-152-029-MY4). The wider project this study was part of was funded by the New Zealand Ministry of Education.

## Conflict of Interest

The authors declare that the research was conducted in the absence of any commercial or financial relationships that could be construed as a potential conflict of interest.

## Publisher’s Note

All claims expressed in this article are solely those of the authors and do not necessarily represent those of their affiliated organizations, or those of the publisher, the editors and the reviewers. Any product that may be evaluated in this article, or claim that may be made by its manufacturer, is not guaranteed or endorsed by the publisher.
